# Post-ictal psychosis: A case report

**DOI:** 10.1192/j.eurpsy.2021.1707

**Published:** 2021-08-13

**Authors:** M. Turki, R. Ouali, S. Ellouze, R. Charfi, W. Abid, T. Babbah, N. Halouani, J. Alouou

**Affiliations:** Psychiatry “b” Department, Hedi Chaker University hospital, sfax, Tunisia

**Keywords:** post-ictal psychosis, Epilepsy

## Abstract

**Introduction:**

People suffering from chronic diseases, especially epilepsy, are more likely to suffer from neurobehavioral disorders, like psychotic states. Postictal psychosis (PIP) is one of these potentially serious complications, that classically follows exacerbations of seizures.

**Objectives:**

The present paper aimed to study the clinical and therapeutic aspects of PIP.

**Methods:**

We report a case of PIP, which involved a patient hospitalized in psychiatry department, and discuss it in light of the relevant literature.

**Results:**

We report the case of a 27-year-old man, with medical history of generalized epilepsy which was well stabilized under treatment (carbamazepine 600 mg/day). The patient was hospitalized for dangerous behaviors after having experienced 2 episodes of seizure activity in context of poor therapeutic adherence. Psychiatric assessment revealed a psychomotor instability, a pressured speech and hallucinatory behavior. There were no delirium symptoms. Neurological examination showed no localization signs, and cerebral imaging was normal. The patient was treated with benzodiazepines (Diazepam), associated to antipsychotics (Haloperidol). His antiepileptic drug was quickly reintroduced. After 48 hours of treatment, psychiatric symptoms improved. The patient returned to its baseline condition after 7 days.

**Conclusions:**

The short-term prognosis of PPI is often favorable, compared to other psychotic disorders. However, more severe psychiatric disorders can potentially develop in the long-term, raising diagnostic and therapeutic difficulties. Thus, a good collaboration between psychiatrists and neurologists is highly desirable in order to better adapt the treatment.

**Disclosure:**

No significant relationships.

